# Supplementary material for: Probing spectral features of quantum many-body systems with quantum simulators
Source: Nat Commun. 2025 Feb 6;16:1403. doi: 10.1038/s41467-025-55955-2 (PMC11803105; doi:10.1038/s41467-025-55955-2)
Supplement: Supplementary file 1 — Supplementary Information [file 41467_2025_55955_MOESM1_ESM.pdf]

Supplementary Information for "Probing spectral features of quantum many-body systems with quantum simulators"

Jinzhao Sun,[1,2,*] Lucia Vilchez-Estevez,[1,†] Vlatko Vedral,[1] Andrew T. Boothroyd,[1] and M. S. Kim[2]

[1]*Clarendon Laboratory, University of Oxford, Parks Road, Oxford OX1 3PU, United Kingdom*
[2]*Blackett Laboratory, Imperial College London, London SW7 2AZ, United Kingdom*

(Dated: December 10, 2024)

I. METHODS

This section elaborates on the framework and the spectroscopic method developed in the main text. In Section I A, we first prove that the two spectroscopic methods which introduce τ in different ways are equivalent. In Section I B, we analyse the mechanism by which our spectroscopy method is capable of detecting spectral properties. In Section I C, we study the estimation error of the transition energies under the finite circuit depth and sampling numbers, and provide the computational complexity of our method. In Section I D, we analyse the effect of algorithmic errors on our method.

A. The spectral detector and the equivalence of the two formalisms

In the main text, we consider $\tilde{g}(t) := \int_{-\infty}^{+\infty} p(\omega)e^{i\omega t}d\omega$ and its dual form $p(\omega) = \frac{1}{2\pi}\int_{-\infty}^{+\infty} \tilde{g}(t)e^{-i\omega t}dt$. The normalised function $g(t)$ is then introduced with the normalisation factor $c := \int_{-\infty}^{+\infty} |\tilde{g}(t)|dt$ and the phase factor $e^{i\theta_t} := \tilde{g}(t)/(cg(t))$. We have $g(t) = \frac{1}{c}\int_{-\infty}^{+\infty} p(\omega)e^{-i\theta_t}e^{i\omega t}d\omega$ and the dual form $p(\omega) = \frac{c}{2\pi}\int_{-\infty}^{+\infty} g(t)e^{i\theta_t}e^{-i\omega t}dt$. If τ is introduced as a separate parameter that is irrelevant to the Fourier transform, it is straightforward to have

$$\tilde{g}_\tau(t) := \int_{-\infty}^{+\infty} p_\tau(\omega)e^{i\omega t}d\omega \tag{1}$$

and

$$p_\tau(\omega) = \int_{-\infty}^{+\infty} \tilde{g}_\tau(t)e^{-i\omega t}dt. \tag{2}$$

On the other hand, we can arrange that τ is coupled with ω in the Fourier transform, which is the way introduced in the main text. Then we have

$$g(t) = \frac{1}{c}\int_{-\infty}^{+\infty} p(\tau\omega)e^{-i\theta_t}e^{i\tau\omega t}d(\tau\omega) \tag{3}$$

and

$$p(\tau\omega) = \frac{c}{2\pi}\int_{-\infty}^{+\infty} g(t)e^{i\theta_t}e^{-i\tau\omega t}dt. \tag{4}$$

The following derivation shows the equivalence of these two ways. According to Eq. (1), $\tilde{g}_\tau(t)$ takes the form of

$$\begin{aligned}\tilde{g}_\tau(t) &= \int_{-\infty}^{+\infty} p(\tau\omega)e^{i\omega t}d\omega \\ &= \frac{1}{\tau}\int_{-\infty}^{+\infty} p(\tau\omega)e^{i\omega\tau\frac{t}{\tau}}d(\tau\omega) = \frac{1}{\tau}\tilde{g}(\frac{t}{\tau}).\end{aligned} \tag{5}$$

* jinzhao.sun.phys@gmail.com
† lucia.vilchezestevez@physics.ox.ac.uk

The normalisation factor of $\tilde{g}_\tau(t)$ is given by

$$c(\tau) := \int_{-\infty}^{+\infty} |\tilde{g}_\tau(t)| dt = \int_{-\infty}^{+\infty} \frac{1}{\tau} |\tilde{g}(\frac{t}{\tau})| dt = c. \tag{6}$$

We define a normalised function as

$$\Pr(t,\tau) := \frac{|\tilde{g}_\tau(t)|}{c(\tau)} = \frac{1}{c}\frac{1}{\tau}|\tilde{g}(\frac{t}{\tau})| = \frac{1}{\tau} g(\frac{t}{\tau})$$

which can be expressed by $\Pr(t,\tau) = \frac{1}{c(\tau)} \int_{-\infty}^{+\infty} p_\tau(\omega) e^{-i\theta_t} e^{i\omega t} d\omega$, and the dual form is given by

$$p(\tau\omega) = \frac{c(\tau)}{2\pi} \int_{-\infty}^{+\infty} \Pr(t,\tau) e^{i\theta_t} e^{-i\omega t} dt. \tag{7}$$

According to Eq. (7), we have

$$\begin{aligned} p(\tau\omega) &= \frac{c(\tau)}{2\pi} \int_{-\infty}^{+\infty} \frac{1}{\tau} g(\frac{t}{\tau}) e^{i\theta_t} e^{-i\omega t} dt \\ &= \frac{c(\tau)}{2\pi} \int_{-\infty}^{+\infty} g(\frac{t}{\tau}) e^{i\theta_t} e^{-i\tau\omega\frac{t}{\tau}} d(\frac{t}{\tau}) \\ &= \frac{c}{2\pi} \int_{-\infty}^{+\infty} g(t') e^{i\theta_{t'}} e^{-i\tau\omega t'} dt'. \end{aligned} \tag{8}$$

Here, we used that $g(t)$ and $g(\tau t)$ have the same phase factor. The above result indicates that Eq. (7) can be converted to Eq. (4) and hence the two formalisms are equivalent.

As discussed in the main text, there are two necessary requirements for inferring the transition energy $\Delta_{n',n} := E_{n'} - E_n$: (1) a sufficiently large coherence $\Gamma_{n',n}$, which can also be regarded a spectral weight and (2) a proper function $p(\omega)$ that ensures that $\Delta_{n',n}$ can be distinguished from other transition energies. The following strategy can be used in order to satisfy the first condition. We first prepare the initial state ρ as the ground state of the noninteracting system governed by H_0. The interaction H_1 at $t = 0$ is then suddenly turned on. The state will be evolved under the Hamiltonian $H = H_0 + H_1$. In a weakly coupled regime, where the initial state ρ is close to the ground state $|0\rangle$ of H, the initial state can be expanded using the first-order perturbation as $\rho \simeq \rho^{00}|0\rangle\langle 0| + \sum_{n\neq 0} \rho^{0n}|0\rangle\langle n| + \rho^{n0}|n\rangle\langle 0|$. The state coherence ρ^{n0} is nonzero, which indicates that a transition between the eigenstate $|n\rangle$ and the ground state $|0\rangle$ is allowed. Therefore, by appropriately choosing the observable, we can in principle detect $\Delta_{n,0}$.

We can also probe the transition energy between the single-particle excited states $|n\rangle$ and $|n'\rangle$ in the weakly coupled system, similar to the way introduced in the main text. The transition could be expressed as $\langle n|\hat{O}|n'\rangle = \langle \mathbf{q}|\hat{O}|\mathbf{q}+\mathbf{k}\rangle$ where we represent the particle excitations in the momentum space, and the momentum selection rule is imposed. If the excitations are restricted to a single-particle manifold, we may choose an observable that conserves the particle number $\hat{O} = \sum_{\mathbf{p},\mathbf{p}'} A_{\mathbf{p},\mathbf{p}'} \hat{\gamma}_{\mathbf{p}}^\dagger \hat{\gamma}_{\mathbf{p}'}$. In this case, the observation is ensured to be nonzero since $\langle n|\hat{O}|n'\rangle = A_{\mathbf{q},\mathbf{q}+\mathbf{k}}$. The derivation of the coherence $\Gamma_{n',n}$ was shown in Methods.

B. Extenstion of spectroscopic methods and the relation to the projection-based methods

To further understand why the engineered spectroscopy methods could be used to select the transition energies, we show that our method is closely related to the spectral-filter-based quantum algorithms, which effectively realise imaginary time evolution and can thus infer the eigenstates and eigenvalues. In this section, we analyse the mechanism by which our spectroscopy method is capable of detecting spectral properties. We will discuss the relation between our method and algorithmic cooling developed in [1].

Recall that we have introduced the function $G(t)$, which can be equivalently written in the Schrödinger picture as

$$G(t) = \langle\psi_0|e^{iHt}\hat{O}e^{-iHt}|\psi_0\rangle\,. \tag{9}$$

This definition of the two-time correlation can be extended as

$$G(t_1,t_2) = \langle\psi_0|e^{iHt_1}\hat{O}e^{-iHt_2}|\psi_0\rangle\,. \tag{10}$$

Let us define a weighted Fourier transform of $G(\tau t, \tau t')$ as

$$G(\omega, \omega') = \frac{c^2}{(2\pi)^2} \int_{-\infty}^{\infty} G(\tau t, \tau t') g(t) g(t') e^{i\theta_t} e^{-i\theta_{t'}} e^{-i\tau\omega t} e^{i\tau\omega' t'} dt dt'. \tag{11}$$

We have

$$G(\omega, \omega') = \sum_{n,n'=0} \Gamma_{n',n} p(\tau(E_n - \omega)) p(\tau(E_{n'} - \omega'))]. \tag{12}$$

Here, the function $p(\tau\omega)$ is the dual Fourier transform of $g(t)$ related by $p(\tau\omega) = \frac{c}{2\pi} \int g(t) e^{i\theta_t} e^{-i\tau\omega t} dt$, which is the same as Eq. (4).

Eq. (12) indicates that the energies of $|n\rangle$ and $|n'\rangle$, which are originally connected by the energy selection rule, are now decoupled. Therefore, one can directly evaluate the energy instead of the energy gaps by tuning the parameters ω, ω'. In particular, when we consider $\hat{O} = I$ and $t' = 0$, where

$$\begin{aligned} G(\omega, 0) &= \frac{c}{2\pi} \int_{-\infty}^{\infty} G(\tau t, 0) e^{-i\tau\omega t} dt. \\ &= \frac{c}{2\pi} \int_{-\infty}^{\infty} \langle \psi_0 | e^{i\tau H t} | \psi_0 \rangle \, g(t) e^{-i\tau\omega t} dt \\ &= \frac{c}{2\pi} \sum_j |c_j|^2 \int_{-\infty}^{\infty} g(t) e^{i\tau(E_j - \omega)t} dt \\ &= \sum_j |c_j|^2 p(\tau(E_j - \omega)). \end{aligned} \tag{13}$$

To see why $G(\omega, 0)$ could select the eigenvalues, let us consider a matrix function acting on the Hamiltonian as

$$\hat{p}(H) := \sum_{i=0}^{N-1} p(E_i) \left| u_i \right\rangle \left\langle u_i \right|, \tag{14}$$

where $p(h): \mathbb{R} \to \mathbb{C}$ is a generic continuous-variable function determining the transformation of the spectrum of the Hamiltonian. One can verify that

$$G(\omega, 0) = \langle \psi_0 | \hat{p}(\tau(H - \omega)) | \psi_0 \rangle, \tag{15}$$

which indicates that $G(\omega, 0)$ effectively realises the spectral filter operator $\hat{p}$ on the initial state. For instance, the projection operator could be $\hat{p}(\tau H) = e^{-\tau^2 H^2}$, which projects out the contribution of other eigenstates with an increasing τ.

The eigenvalue information associated with the initial state $|\psi_0\rangle$ be expressed by

$$P(\omega) = \sum_j |c_j|^2 \delta(\omega - E_j), \tag{16}$$

and one can verify that

$$G(\omega, 0) = (p \star P)(\omega), \tag{17}$$

which is because

$$(p \star P)(\omega) = \int_{-\infty}^{\infty} p(t) P(\omega - t) dt = \sum_j |c_j^2| \int_{-\infty}^{\infty} \delta(\omega - E_j - t) dt = \sum_j |c_j|^2 p(E_j - \omega). \tag{18}$$

Note that the method in [2] could be regarded as a special case of our method when taking the filter operator to be an identity. They mainly discussed the dynamics simulation (rather than the spectroscopic feature estimation) within a linear response framework. Beyond the linear response regime, our method could be applicable to detect nonlinear spectroscopic features. Spectroscopic signatures appearing in nonlinear response can be found in [3]. For example, we can apply perturbations three times and obtain the higher order time correlation functions, similarly to the 2D coherent spectroscopy. We can resolve the continuum of the excitation spectrum by analysing the nonlinear susceptibility [4]. It is worth noting that their method for simulating the dynamics of bosonic and fermionic systems could be employed in this context.

C. Analysis of the algorithmic error and resource requirement

In this section, we provide proof for Lemma 1 and Lemma 2 in Methods.

Proof. (of Lemma 1)
When ω is close to Δ_j satisfying $|\omega - \Delta_j| \leq 0.5\varepsilon$, we have

$$\Gamma_j - G(\omega) \leq (1 - p(\tau(\Delta_j - \omega)))\Gamma_j + \max_{i \neq j} p(\tau(\Delta_i - \omega)) \leq (1 - e^{-\tau^2(0.5\varepsilon)^2})\Gamma_j + 0.05\tau^2\varepsilon^2\Gamma_j \leq 0.3\tau^2\varepsilon^2\Gamma_j. \tag{19}$$

The second inequality uses the following fact that the inequality

$$\max_{i \neq j} p(\tau(\Delta_i - \omega)) \leq e^{-\tau^2(0.9\gamma)^2} \leq 0.05\tau^2\varepsilon^2\Gamma_j, \tag{20}$$

holds when $\tau = \frac{1}{0.9\gamma}\sqrt{\ln\left(\frac{20}{\varepsilon^2\Gamma_j}\right)} \geq 1$ and $\varepsilon \leq 0.2\gamma$. Thus the first inequality in Eq. (14) in Lemma 1 holds.

On the other hand, the quantity $\Gamma_j - G(\omega)$ can be lower bounded by

$$\begin{aligned} \Gamma_j - G(\omega) &\geq (1 - p(\tau(\Delta_j - \omega)))\Gamma_j - \max_{i \neq j} p(\tau(\Delta_i - \omega)) \\ &\geq (1 - e^{-\tau^2\varepsilon^2})\Gamma_j - e^{-\tau^2(0.9\gamma)^2} \\ &\geq 0.85\tau^2\varepsilon^2\Gamma_j - 0.05\tau^2\varepsilon^2\Gamma_j = 0.8\tau^2\varepsilon^2\Gamma_j. \end{aligned} \tag{21}$$

In the second inequality, we used the fact that $|\Delta_i - \omega| \geq 0.9\gamma$ for $i \neq j$. The third inequality $e^{-\tau^2\varepsilon^2} \leq 1 - 0.85\tau^2\varepsilon^2$ holds when $\tau\varepsilon < 1/2$, which can be achieved when $\varepsilon \leq \tilde{\mathcal{O}}(\gamma(\ln{\Gamma_j}^{-1})^{-\frac{1}{2}})$.

□

Lemma 1. *The estimation $\hat{\Delta}_j$ is related to the true transition energy Δ_j by $|\hat{\Delta}_j - \Delta_j| \leq \varepsilon$. with a failure probability of δ, when $\tau \geq \frac{1}{0.9\gamma}\sqrt{\ln\left(\frac{10}{\varepsilon^2\Gamma_j}\right)}$, the cutoff $T \geq 2\sqrt{2\ln(\sqrt{10}\tau^{-1}\varepsilon^{-1})}$ and the number of measurements $N_s \geq 200(\varepsilon^4\Gamma_j^2\tau^4)^{-1}\ln(4/\delta)$.*

Proof. Suppose we already have a rough estimation of Δ_j which lies in the range of $[\Delta_j^L, \Delta_j^R]$ with $\Delta_j^L = \Delta_j - \gamma/2$ and $\Delta_j^R = \Delta_j + \gamma/2$. Same as that in [1, 5], we consider a uniformly discretised grid with the grid resolution at least ε. With this consideration, the discredited frequency is given by $\omega_k = \Delta_j^L - 0.5\gamma + \frac{\gamma(k-1)}{M}$ for $k = 1, 2, ..., M$ with $M = 2\lceil\gamma/\varepsilon\rceil + 1$ to ensure the resolution. It is worth noting that computing $\hat{G}^{(T)}(\omega)$ with different values of ω_k requires purely classical computing, and M is irrelevant to the measurement numbers N_s. One can use a much smaller grid size without demanding any additional quantum measurements.

The following shows that the transition energy can be estimated within a certain precision given the resources listed in Lemma 1. With the grid defined above, the estimation is determined by

$$\hat{\Delta}_j = \mathrm{argmax}_k \hat{G}^{(T)}(\omega_k). \tag{22}$$

The question is whether the estimation has a bounded error satisfying $|\hat{\Delta}_j - \Delta_j| \leq \varepsilon$.

The error due to a finite number of measurements can be bounded using the Hoeffding inequality. The estimator $\hat{G}^{(T)}(\omega)$ is related to ideal $G^{(T)}(\omega)$ by

$$|G^{(T)}(\omega) - \hat{G}^{(T)}(\omega)| \leq \Gamma_j\varepsilon_n, \ \forall\omega \in \mathbb{R} \tag{23}$$

with a failure probability $\delta/2$ when $N_s = 2(\varepsilon_n\Gamma_j)^{-2}\ln(\frac{4}{\delta})$. Here we used the fact that ${G_i}^{(T)}(\omega) \leq 1$. Since the grid resolution is $\varepsilon/2$, there exists k_m, such that

$$|\omega_{k_m} - \Delta_j| \leq 0.5\varepsilon. \tag{24}$$

Combining the truncated spectral detector Eq. (16) (defined in Methods) and Eq. (23), we have

$$\begin{aligned} \Gamma_j - \hat{G}^{(T)}(\omega_{k_m}) &\leq (\Gamma_j - G(\omega_{k_m})) + |G(\omega_{k_m}) - G^{(T)}(\omega_{k_m})| + |G^{(T)}(\omega_{k_m}) - \hat{G}^{(T)}(\omega_{k_m}))| \\ &\leq 0.3\tau^2\varepsilon^2\Gamma_j + \varepsilon_T\Gamma_j + \Gamma_j\varepsilon_n \leq 0.5\tau^2\varepsilon^2\Gamma_j \end{aligned} \tag{25}$$

with a failure probability $\delta/2$. In the last inequality, we have set $\varepsilon_n = 0.1\tau^2\varepsilon^2$ and $\varepsilon_T \leq 0.1\tau^2\varepsilon^2$. The latter condition can be satisfied by setting $T \geq 2\sqrt{2\ln(\sqrt{10}\tau^{-1}\varepsilon^{-1})}$. This indicates that

$$\hat{G}^{(T)}(\hat{\Delta}_j) \geq \hat{G}^{(T)}(\omega_{k_m}) > (1-0.5\tau^2\varepsilon^2)\Gamma_j. \tag{26}$$

On the other hand, if the estimator determined by Eq. (22) fails to give an accurate estimation up to ε, that is, $|\hat{\Delta}_j - \Delta_j| > \varepsilon$, then using Lemma 1 we have $\Gamma_j - G(\hat{\Delta}_j) \geq 0.8\tau^2\varepsilon^2\Gamma_j$. We have

$$\begin{aligned}\Gamma_j - \hat{G}^{(T)}(\hat{\Delta}_j) &\leq (\Gamma_j - G(\hat{\Delta}_j)) + |G(\hat{\Delta}_j) - G^{(T)}(\hat{\Delta}_j)| + (|G^{(T)}(\hat{\Delta}_j) - \hat{G}^{(T)}(\hat{\Delta}_j)| \\ &\geq 0.8\tau^2\varepsilon^2\Gamma_j - \varepsilon_T\Gamma_j - \varepsilon_n\Gamma_j \geq 0.6\tau^2\varepsilon^2\Gamma_j\end{aligned} \tag{27}$$

with a failure probability $\delta/2$. The truncated estimator thus can be bounded

$$\hat{G}^{(T)}(\hat{\Delta}_j) \leq \Gamma_j(1-0.6\tau^2\varepsilon^2), \tag{28}$$

which violates Eq. (26). Consequently, the assumption does not hold, which in turn proves that $|\hat{\Delta}_j - \Delta_j| \leq \varepsilon$ with a failure probability at most δ.

□

D. Coherent error effect in transition energy estimation

Our proposal relies on the measurement of $\hat{O}(t)$, which essentially requires the realisation of e^{-iHt}. The implementation by using product formulae will introduce a coherent Trotter error. By way of illustration, let us consider a simplified lattice model, whose Hamiltonian consists of two non-commutive terms as $H = H_1 + H_2$. The first-order Trotter formula reads

$$S_1(t) = e^{-itH_2}e^{-itH_1} = e^{-itH_{\text{eff}}} \tag{29}$$

where H_{eff} has an explicit form as $H_{\text{eff}} = H + \frac{1}{2}[H_1, H_2] + ...$ by the Baker–Campbell–Hausdorff expansion. We can find that this algorithmic error brings about a perturbation to the original Hamiltonian which can be formally represented as $H_{\text{eff}} = H + \delta H$. The spectral features that we can actually probe are those of the new Hamiltonian.

Let us denote the eigenbases of the new effective Hamiltonian as $\{|\nu\rangle\}$. The quantity $G(\omega)$ becomes

$$G(\omega) = \sum_{\nu',\nu} \Gamma_{\nu',\nu} p(E_{\nu'} - E_\nu - \omega). \tag{30}$$

In the case of a lattice model which preserves the translation invariance, the new Hamiltonian also conserves translation invariance, that is, $\hat{\mathbf{P}}|\nu\rangle = \mathbf{p}_\nu|\nu\rangle$. The observable expectation is given by $\langle\nu|\hat{O}(\mathbf{x})|\nu'\rangle = e^{i(\mathbf{p}_{\nu'}-\mathbf{p}_\nu)\mathbf{x}}\langle\nu|\hat{O}|\nu'\rangle$. In this case, ν' and ν are connected according to the momentum selection rule $\mathbf{k} = \mathbf{p}_{\nu'} - \mathbf{p}_\nu$, which is similar to the noiseless one. However, noise will result in a deviation in transition energies.

It is natural to examine the noise effect using perturbation theory. The eigenenergy has a deviation from the original one $E_{\nu'} = E_n + \delta E_n$ with $\delta E_n = \langle n|\delta H|n\rangle$. The first-order change in the nth eigenstate is related to the unperturbed one by $|\nu\rangle = |n\rangle + \sum_{m\neq n} A_{mn}|m\rangle$ with $A_{mn} = \Delta_{nm}^{-1}\langle m|\delta H|n\rangle$. The quantity $G(\omega)$ becomes

$$G(\omega) = \sum_{\nu',\nu} \Gamma_{\nu',\nu} p(E_{n'} - E_n + \delta E_{n'} - \delta E_n - \omega). \tag{31}$$

The resolved energy difference $E_{n'} - E_n + \delta E_{n'} - \delta E_n$ has a deviation from the original one. Nonetheless, the momentum selection rule still holds, which imposes $\mathbf{k} = \mathbf{p}_{\nu'} - \mathbf{p}_\nu$. Here the coherence $\Gamma_{\nu',\nu}$ is changed from $\Gamma_{n',n}$. Up to the first order, the coherence has a difference as

$$\begin{aligned}\Gamma_{\nu',\nu} &= \left(\rho^{n'n} + \sum_m A_{mn}\rho^{n',m} + \sum_m A^*_{mn'}\rho^{m,n}\right)\left(\langle n|\hat{O}|n'\rangle + \sum_m A_{mn'}\langle n|\hat{O}|m\rangle + \sum_m A^*_{mn}\langle m|\hat{O}|n'\rangle\right) \\ &= \Gamma_{n',n} + \langle n|\hat{O}|n'\rangle\left(\sum_m A_{mn}\rho^{n',m} + \sum_m A^*_{mn'}\rho^{m,n}\right) + \rho^{n'n}\left(\sum_m A_{mn'}\langle n|\hat{O}|m\rangle + \sum_m A^*_{mn}\langle m|\hat{O}|n'\rangle\right)\end{aligned} \tag{32}$$

One can find that in addition to $\Gamma_{n',n}$, some eigenstates $|m\rangle$, which are absent in the original selection rule, also contribute to $G(\omega)$. In the case where translation invariance is broken, the momentum selection rules will be lifted and the dispersion becomes broadened. This is similar to that of disordered systems. We can similarly derive the deviation of the energy and the coherence. We leave more detailed derivation to dedicated readers.

II. NOISE ANALYSIS, MITIGATION AND RESOURCE REQUIREMENTS

As discussed in the main text, the error sources include (1) algorithmic error, (2) uncertainty error due to a finite number of measurements and (3) error due to imperfect quantum operations. The preceding sections have discussed the algorithmic error and the uncertainty error (which is an unbiased error). We have discussed the resource requirement for the circuit depth and the number of measurements to ensure the simulation accuracy up to precision ϵ. In this section, we will analyse the error effect due to device noise and its mitigation strategy.

A. General strategy

We first introduce a general quantum error mitigation (QEM) strategy developed in [6] based on probabilistic error cancellation, and discuss the resource cost when considering noise. In our protocol, we mainly need the implementation of e^{-iHt_i} with different time lengths t_i. With analogue quantum simulators, e^{-iHt_i} is directly implemented through engineering the Hamiltonian of controllable quantum hardware. When assuming a Markovian type of noise that appears due to unwanted coupling between the device and the environment, the time evolution could be described by the Lindblad master equation of the noisy state $\rho_N(t)$

$$\frac{d\rho_N(t)}{dt} = -i[H(t), \rho_N(t)] + \lambda\mathcal{L}\big[\rho_N(t)\big]. \tag{33}$$

In the above equation, $\mathcal{L}[\rho] = \frac{1}{2}\sum_k(2L_k\rho L_k^\dagger - L_k^\dagger L_k\rho - \rho L_k^\dagger L_k)$ represents the noise superoperator with error strength λ that describes the coupling with the environment. Common noise models include dephasing and damping types of noise, which can be described by local Lindblad terms [7, 8].

The main idea of probabilistic error cancellation [9, 10] is to apply a recovery operation to the noisy state, so that the noisy process is mitigated. Below we briefly the probabilistic error cancellation proposed in [6]. Specifically, the noisy evolution of Eq. (33) can be represented as $\rho_N(t+\delta t) = \mathcal{E}_N(t)\rho_\alpha(t)$ within a small time step δt where $\mathcal{E}_\alpha(t)$ denotes the noisy channel within small δt. A recovery operation $\mathcal{E}_Q$ is an operation that can approximately map the noisy evolution back to the noiseless one up to the first order as $\mathcal{E}_I(t) = \mathcal{E}_Q\mathcal{E}_N(t) + \mathcal{O}(\delta t^2)$. We can adopt a probabilistic way to effectively realise $\mathcal{E}_Q$. That is, we can decompose $\mathcal{E}_Q$ into a linear sum of physical operators $\{\mathcal{B}_j\}$ (i.e. basis operators) $\mathcal{E}_Q = c\sum_j \alpha_j p_j \mathcal{B}_j$, with coefficients $c = 1 + \mathcal{O}(\lambda\delta t)$, $\alpha_j = \pm 1$, and a normalised probability distribution p_j. The cost is $\mathcal{O}(\exp(\lambda T_{\max})$, and thus, the error mitigation is efficient as long as $\lambda T_{\max}$ is bounded. We could see that in order to effectively implement QEM, the total required time should be short – if the maximum time is short, then the number of measurements can be bounded. As the time complexity of our method is $\mathcal{O}(\log(1/\epsilon))$, the total running time is $\mathcal{O}(\mathrm{poly}(1/\epsilon))$.

For a short time evolution, the QEM strategy can be run in a stochastic setting, and it works for both analogue and digital quantum simulators. The number of the basis operations required for performing error mitigation on average scales linear in time as $\mathcal{O}(\lambda T_{\max})$.

B. Error mitigation for global depolarising noise

Below, we use a simple example to illustrate the error mitigation process. In order to mitigate the error effect, we need to assume the noise type. Within a short time, a simple way is to consider a depolarising noise model: the state remains unaffected with probability $\lambda\delta t$ while becomes a mixed state with probability $1-\lambda\delta t$, with noise strength characterised by λ. This noisy process can thus be described by

$$\mathcal{E}_{\delta t}(\rho) = (1-\lambda\delta t)\rho + \lambda\delta t\rho_{\mathrm{mix}} \tag{34}$$

where $\rho_{\mathrm{mix}} = \frac{I}{2^N}$ is the maximally mixed state and I is the identity matrix as defined in the main text. We could see that the noisy time-evolved state can be described by

$$\rho(t) = \prod_{\delta t}^{T}(\mathcal{E}_{\delta t}\circ\mathcal{U}_{\delta t})(\rho) = \Lambda(t)^{-1}\mathcal{U}_t(\rho) + (1-\Lambda(t)^{-1})\rho_{\mathrm{mix}} \tag{35}$$

where we have defined $\mathcal{U}_t := U(\cdot)U^\dagger$ and $\Lambda(t) = e^{\lambda t}$. The overall action can be described by

$$\mathcal{E}\circ\mathcal{U}_t(\rho) = \Lambda(t)^{-1}U_t\rho U_t^\dagger + (1-\Lambda(t)^{-1})\rho_{\mathrm{mix}} \tag{36}$$

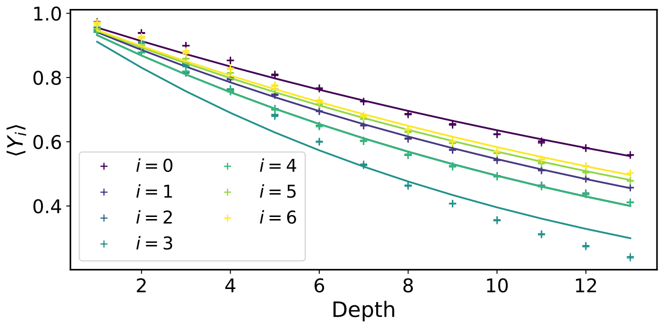

Supplementary Figure 1. Exponential fitting for individual qubits in the 7-site Ising model with local depolarising noise. Noise strength is set to $p = 0.005$. The setup is the same as that in Figure 5 in the main text.

This can be understood by evolving the state under $\mathcal{U}$ followed by a noise channel $\mathcal{E}(\cdot) = \Lambda(t)^{-1}(\cdot) + (1 - \Lambda(t)^{-1})\rho_{\text{mix}}$. The effective action of such a noise channel is a global depolarising noise. We can find the noise channel coupled with the unitary operator is only dependent on t. The expectation value of a Pauli operator is

$$\mathbb{E}\hat{o}_{noisy}(t) = \text{Tr}(\mathcal{E} \circ \mathcal{U}_t(\rho)) = \Lambda(t)^{-1}\mathbb{E}\hat{o}_{ideal}(t). \tag{37}$$

.

As discussed in the main text, we can determine λ by fitting the noisy results with the ideal ones, in a similar spirit of randomised benchmarking. We run the circuit $(\tilde{\mathcal{U}}_{t/m} \circ \mathcal{U}_{t/m})^m = \mathcal{I}$ where $\tilde{\mathcal{U}}_{t/m} = U^\dagger(t/m)(\cdot)U(t/m)$, which should be an identity channel in the ideal case. In the presence of noise, it becomes

$$(\mathcal{E}_{t/m} \circ \tilde{\mathcal{U}}_{t/m} \circ \mathcal{E}_{t/m} \circ \mathcal{U}_{t/m})^m(\rho) = \Lambda(2t)^{-1}\rho + (1 - \Lambda(2t)^{-1})\rho_{\text{mix}} \tag{38}$$

when the noise is gate-independent. We could run it by fitting the result undergoing by the channel $(\mathcal{E}_{t/m} \circ \tilde{\mathcal{U}}_{t/m} \circ \mathcal{E}_{t/m} \circ \mathcal{U}_{t/m})^m$ with different t and m, such that this fitting process could be robust against state preparation and measurement errors.

Recall that each $\hat{o}(\tau t_i)$ is measured on a quantum computer. When doing error mitigation by scaling, the variance of $G(\omega)$ is amplified by factor $\Lambda(\tau|t_i|)$. In order to suppress the statistical error to ϵ, the number of measurements is amplified by the factor. The variance is related to $\Lambda(\tau \max_i |t_i|)$. Therefore, when the time is $\mathcal{O}(\log(1/\epsilon))$, the total time is $\mathcal{O}(\text{poly}(1/\epsilon))$. For other methods with the maximum time complexity being $\mathcal{O}(\text{poly}(1/\epsilon))$, the total time complexity is $\mathcal{O}(\exp(1/\epsilon))$.

It is worth noting that the above process can be regarded as an effective noise mitigation at the algorithmic level. This is in contrast to the case of general noise discussed in Section II A where physical operations (chosen from the set $\{\mathcal{B}_j\}$ are required to mitigate the noise.

C. Numerical simulation

In this section, we provide additional simulation results to support the noise mitigation strategies proposed in the main text. Specifically, we display fittings results for each qubit, results for different noise rates, and results for larger system sizes.

In the main text, we demonstrated that the average expectation value $\sum_i \langle Y_i \rangle$ can be approximated by an exponential decay function. In Supplementary Figure 1, we present the fitting results for the individual expectation value in the 7-site Ising model with $h_z = 0.1$. For each qubit i, the observable $\langle Y_i \rangle$ is fitted using an exponential decay function, with high predictive power $R \geq 0.98$. These individual fittings serve as the basis for our error mitigation strategy, showing that each qubit noisy measurement outcomes can be corrected effectively using these exponential functions. We note that this fitting strategy may be less effective for more general types of noise, and thus, we need error mitigation to obtain reliable results.

In Supplementary Figure 2, we show the results for the same 7-site Ising model with a lower noise rate $p = 0.001$. The figure presents the time dynamics and frequency domain analysis under different conditions: noisy, error mitigated, and ideal (noiseless). The QEM protocol continues to perform well in mitigating the noise effect, with observable errors reduced both in the time and frequency domains. These results indicate that our spectroscopic protocol maintains effective across different noise rates. To validate the scalability of our method, Supplementary

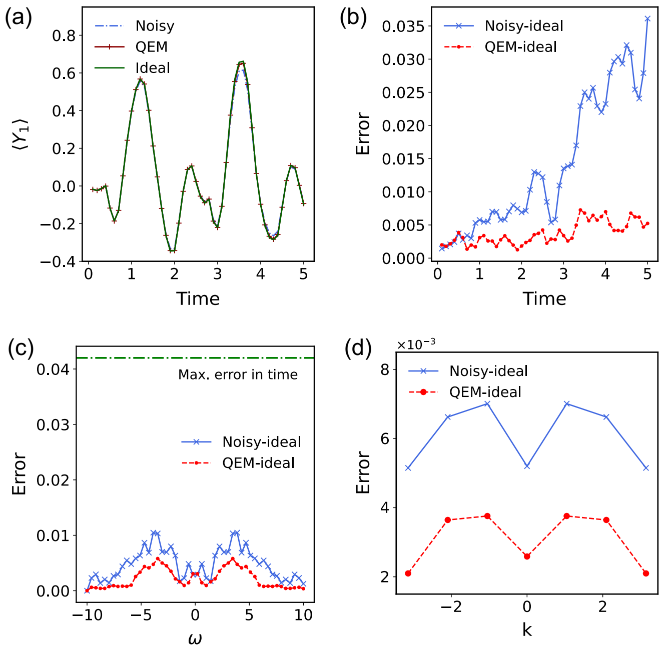

Supplementary Figure 2. Noisy and error-mitigated results for spectral property estimation. (a) Time evolution of the observable $\langle Y_i \rangle$ at site $i = 1$. The figure shows the results of the noisy (with noise rate $p = 0.001$), the error mitigated and the ideal (noiseless) cases for a 7-site Ising model. (b) RMSE of the noisy and the error-mitigated results in the time domain. (c) Error of the noisy and error mitigated spectrum in the frequency domain. The green line represents the maximum value of the error in the time domain to compare both results. (d) Error in the k-space.

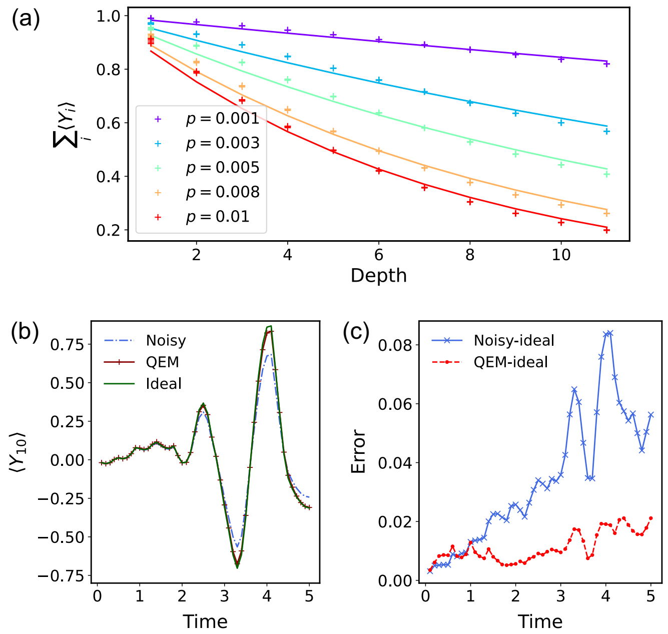

Supplementary Figure 3. Results of the error mitigation on an 11-site Ising model. (a) Average case fitting to exponential function with predictive power $R = 0.99$ for five different noise rates. (b) Time evolution of the observable $\langle Y_i \rangle$ at site $i = 10$. In this case, the noise rate is set at $p = 0.004$. (c) RMSE of the noisy and error mitigated results during the time evolution.

Figure 3 shows the results for an 11-site Ising model with noise rate $p = 0.004$. We follow the same procedure as outlined above. The fitting (a), time dynamics (b) and time domain errors (c) are shown in Supplementary Figure 3

with the noisy, error-mitigated, and ideal cases. The results highlight that the QEM approach continues to be effective even for relatively larger system sizes.

III. COMPARISON WITH OTHER RELATED WORKS

Probing the spectral features of quantum systems is a highly active area of research that has attracted increasing attention. Numerous studies have been conducted in this direction. In this section, we compare our work with several representative studies in the field.

To begin with, we summarise the key elements of our method. This work concerns the central components in probing the transition energies and excitation spectra. The key elements in the spectroscopic method include how the initial state and the observable can be found to observe the transition energies, and whether spectral features can be virtually probed within a short time length. For the first one, we consider an initial state that contains a branch of excitations that we want to probe and we require a nonvanising $\langle n|\hat{O}|n'\rangle$ to observe the transition between $|n\rangle$ and $|n'\rangle$. As widely accepted in the community, these requirements in general remain a challenge, though we have provided a few insights into finding observables in typical quantum systems. Additionally, as the measurement complexity in the estimation of multiple observables can be reduced by using classical shadow methods, we can extract the spectral information from a large number of observables, which is the key idea in [11]. For the second one, we have revealed a close relation between our method and the spectral filter methods, which naturally select the eigenenergies by projecting out the contributions from other eigenstates. Below we discuss related works from different aspects.

A. Scattering spectroscopy experiments and simulation of the experiments

It is interesting to discuss the similarities and differences between our method and conventional spectroscopy techniques. Both are capable of obtaining the energy excitation spectrum of a quantum system, but are different from operational perspectives. We first provide a brief introduction to spectroscopy. Experimental spectroscopy is a state-of-the-art probe approach that is used to uncover complex quantum many-body behaviours. In magnetic neutron scattering, for example, neutrons interact with spins of electrons, and the intensity of the scattered neutrons reflects the magnetic response of electrons in the materials. This in turn carries certain information about the magnetic interaction in the materials being probed. The observable in inelastic neutron scattering [12, 13] is the dynamical structure factor $S(\mathbf{Q},\omega)$, also known as the magnetic response function, which is related to a two-point unequal-time correlator

$$C(t,t') = \langle \hat{\mathbf{S}}_1(t)\hat{\mathbf{S}}_2(t')\rangle\,, \tag{39}$$

by the Fourier transform, where $\hat{\mathbf{S}}$ is a spin operator. In conventional spectroscopy, the observables are related to two-point unequal-time correlation functions $C(t,t')$ and the initial state is in its equilibrium. The two-point unequal-time correlator contains spectral information on the spin dynamics of a many-body system. In a translationally invariant system, the dynamical structure factor $S(\mathbf{Q},\omega)$ reaches its local maximum when the energy selection rule, as well as the momentum selection rule, are both satisfied. In a spectroscopic experiment, consequently, we usually track the peak of intensities in the neutron scattering spectrum, from which we can infer the energy dispersion. Some degrees of freedom in engineering the system are possible in spectroscopy experiments, such as through the application of an external electric or magnetic field. Nevertheless, the cost is huge because of the extreme experimental conditions and the high requirements in synthesising pure materials (pure in the sense that there are not many purities and the interactions types are clear).

Several works considered simulations of spectroscopy [14, 15], which is based on the simulation of the two-point unequal-time correlation function given by Eq. (39). A drawback of the simulated spectroscopy is that it inherits the limitation of spectroscopy experiments and does not overcome it. In addition, in order to measure the unequal-time correlation function, a Hadamard-test circuit (a controlled time evolution) is usually required. As mentioned before, in the spectroscopy experiment or its simulation, the samples to be probed are in their equilibrium state, and thus, the information is restricted to the diagonal form. An advantage of our method is that because our initial state is not a steady state, and thus it can probe the energy difference between different excited states. In terms of implementation, they need one ancilla qubit that controls the rest of the qubits. In contrast, there is no overhead in compiling the non-local gate in our protocol and thus for lattice models, the depth within each time step is $\mathcal{O}(1)$.

In our work, the transition between different excited states can be probed given that the coherence is nonzero. One contribution of this work is to analyse how to choose the initial state and the observables in a physics-inspired way.

This is rarely discussed in existing works. For example, in a recent paper [2], they assumed that the ground state could be prepared as the initial state, although they argued that this is not the scope of their work. As indicated by the complexity conjecture, the ground state and the thermal state are hard to prepare.

Our method is not restricted to the implementation in an analogue way. Indeed, our method is more versatile and can be useful when FTQC is advent. The reason why the method developed in this work can go beyond pure analogue quantum simulation is twofold. The first is about the initial state preparation, and the second one is the the versatility in the time evolution. To see this point concretely, we give a class of examples of which analogue quantum computers may be hard to probe. Let us start with a model Hamiltonian for superconductivity. Because of the large numbers of particles involved, the fluctuations in the number of Cooper pairs should be small, which suggests a mean-field approximation to the BCS Hamiltonian. The BCS Hamiltonian becomes quadratic, which reads

$$H = \sum_{k,\sigma} \epsilon_k \hat{c}^\dagger_{k,\sigma} \hat{c}_{k,\sigma} + \sum_k \left(\Delta_k \hat{c}^\dagger_{k,\uparrow} \hat{c}^\dagger_{-k,\downarrow} + \Delta^*_k \hat{c}_{-k,\downarrow} c_{k,\uparrow} \right) \tag{40}$$

where the irrelevant constant is removed. Analogue simulators can hardly simulate this type of Hamiltonians (e.g. its time evolution), which do not conserve particle numbers. However, since it is bi-linear in terms of creation and annihilation operators, the Hamiltonian can be diagonalized. Specifically, by using a Bogoliubov transformation, this Hamiltonian can be transformed into a diagonal form $H = \sum_{k,\sigma} \omega_k \hat{\gamma}^\dagger_{k\sigma} \hat{\gamma}_{k\sigma}$ where $\hat{\gamma}^\dagger_{k\sigma}$ and $\gamma_{k\sigma}$ are a new set of fermionic operators that satisfy the canonical anticommutation relations, and can be regarded as a rotated basis with respect to the original one. The rotated basis is related to the original basis by the unitary transformation as

$$\hat{\gamma}^\dagger_j = U \hat{c}^\dagger_j U^\dagger \tag{41}$$

where $j = (k, \sigma)$ and U is a unitary operator which does not conserve particle numbers. As discussed in quantum computing literature by Google's team [16], this unitary operator can be decomposed into local operators and thus can be implemented easily on quantum computers.

For the interacting case, we can prepare the initial state with a single quasiparticle excitation,

$$|\psi_0\rangle = \hat{\gamma}^\dagger_j |\text{vac}\rangle = U \hat{c}^\dagger_j |\text{vac}\rangle \, . \tag{42}$$

Although $\hat{\gamma}$ is not the previous operator, it can be implemented with a unitary transformation to the original basis. They also satisfy the canonical anticommutation relations. To prepare the initial state given by Eq. (42), we only need to apply a unitary operator to an easy-to-prepare state. Eq. (42) could serve a good initial state when the quasiparticle picture still holds. The Hamiltonian evolution can be realised by using Trotterisation or a random sampling way that is introduced in the main text.

Our method can be used to simulate the features of linear response, which is one application of the method presented here. The dynamics simulation methods for bosonic and fermionic systems introduced in [2] can be directly employed within our framework. Beyond the scope discussed in [2], we have discussed the conditions for designing the initial state and the complexity of exploring the spectral features.

B. Engineered spectroscopy methods

Finally, we compare our method to engineered spectroscopy methods, in particular Refs. [17–19]. It has been shown that nonequilibrium dynamics after a global quench [17, 20] or a local quench [21] could unveil certain excitation spectra, which has been termed quench spectroscopy. The basic idea is that quench will drive the initial stationary state out of equilibrium and generate low-lying quasiparticle excitations. The excitation spectrum can thus be obtained by measuring a properly chosen observable. For instance, the basic protocol of local quench spectroscopy for a lattice model with translation invariance is that we first initialise the system in its ground state, then apply a local operation to a single lattice site, and finally measure the dynamics of a local observable, which was initially proposed in [21]. It is worth noting that the 'local quench' in the original paper may stretch the conventional meaning of quench. Quench usually refers to a process where parameters in the Hamiltonian are changed in time, and usually the time scale for the change of parameters is very fast. For example, a system is prepared as an eigenstate of a Hamiltonian H_0 at $t < t_0$, while at time t_0, the system is evolved dynamically under a different Hamiltonian $H_0 + H_1$. A more accurate description of 'local quench' in the protocol in [21] could be 'local perturbation'.

Let us consider a one-dimensional transverse field Ising model with,

$$H = \sum_{i<j\leq N} J_{ij} \hat{\sigma}^x_i \hat{\sigma}^x_j + h \sum_{j \leq N} \hat{\sigma}^z_j, \tag{43}$$

where $\hat{\sigma}_i^\alpha$ ($\alpha = x, y, z$) is a Pauli operator on the ith site, J_{ij} is the strength of spin-spin coupling between the ith and jth site. Ref. [17] considered a strong field case $h \gg \max J_{ij}$, in which the energy spectrum of H is split into $N+1$ decoupled subspaces spanned by different excitation numbers. Below we briefly review the proposal in [17]. The Hamiltonian H conserves the total excitations numbers $\hat{n} = \sum_j (\hat{\sigma}_j^z + 1)/2$. They proposed observation of quasiparticle spectroscopy by engineering the initial state consisting of the particular quasiparticle excitations. More specifically, by rotating the spins on each site $|\theta_j\rangle = \cos(\theta_j)|0\rangle_j + \sin(\theta_j)|1\rangle$ where $|0\rangle_j$ represents a spin-up state, the initial state $|\psi_0\rangle = \otimes_{j=1}^N |\theta_j\rangle$ could be a good approximation of a superposition of the ground state and the eigenstate of H in the single-excitation subspace. To probe the single quasiparticle excitations E_k, the initial state is prepared as

$$|\psi_0\rangle \approx |0\rangle + \beta |k\rangle , \tag{44}$$

where β is a small constant, $|0\rangle$ is the ground state and $|k\rangle$ is the eigenstate with a momentum k. Specifically, as suggested in [17], the eigenstates can be written as $|k\rangle = \sum_{j=1}^N \tilde{A}_j^k |1\rangle_j \otimes_{i\neq j} |0\rangle_i$ where for nearest-neighbour couplings $\tilde{A}_j^k = \sqrt{2/(N+1)}\sin(kj\pi/(N+1))$. By setting $\theta_j = \tan^{-1}(\beta \tilde{A}_j^k)$, we have a tensor product state that is a good approximation of Eq. (44). It is easy to verify that the state coherence $\langle 0|\psi_0\rangle \langle\psi_0|k\rangle = \beta$. For probing transitions between $|k\rangle$ and $|k'\rangle$, the authors prepared the state as

$$|\psi_0\rangle \approx |0\rangle + \beta(|k\rangle + |k'\rangle) , \tag{45}$$

in which the state coherence $\langle k|\psi_0\rangle \langle\psi_0|k'\rangle = \beta^2$. The choice of the initial states and observables is a special case of our method as discussed in Methods.

Yoshimura *et al.* considered a time-dependent field $B = B(t)$, which is decreased from a large polarising field to a constant, to create excitations, a method termed diabatic-ramping spectroscopy [18]. The transition energies can be obtained by taking the Fourier transform of the observable dynamics. Senko *et al.* considered a similar strategy, which applies time-dependent field $B(t) = B_0 + B_p \sin(2\pi v_p t)$ for probing the energy spectrum of a weakly coupled system. At the basis of this method is the emergence of an energy resonance between $|n\rangle$ and $|n'\rangle$ when the frequency of the external field, v_p, matches the transition energies $|\Delta_{n',n}|$ [19]. The emergence of resonance at $v_p = |\Delta_{n',n}|$ could be understood by time-dependent perturbation theory. In addition to the above specific Ising model, spectroscopy protocols have demonstrated that excitations can be effectively created in cases of Bose-Hubbard models [20, 22], spin chains [17–19], topological systems [3], and disordered systems [23].

C. Quantum algorithms for eigenenergy estimation

Existing universal quantum algorithms for finding eigenstates and the associated eigenenergies [24–29], such as quantum phase estimation and quantum signal processing, generally require a deep circuit with long-time controlled operations, which remains a challenge for near-term quantum hardware. More importantly, simply in terms of efficiency, having an experiment-friendly method to access the behaviours of materials without requiring too many experimental resources is desirable. Our method only requires the realisation of time evolution e^{-iHt} without reliance on any ancillary qubits, a basic and most promising application of quantum computing [30]. This is in contrast to many Hamiltonian simulation algorithms and variational dynamics simulations, which usually require controlled-unitary operations. Our method is therefore compatible with an analogue quantum simulator, and has the advantage of potentially being more robust against noise. The quantum circuit complexity of our method for transition energy estimation is shown to be logarithmic in precision, while maintaining to be polynomial when device noise is present.

Zintchenko *et al.* [28] proposed an ancilla-free spectral gap estimation method. The spectral gap information is extracted from the computational-basis measurement results $|\langle 0|U^\dagger e^{-i\hat{H}t} U|0\rangle|^2$ with U drawn uniformly from the Haar–random measure, which is different from our work. Recently, Wang *et al.* proposed quantum algorithms for ground state energy estimation [5]. More specifically, they proposed using a Gaussian derivative function in the form of $p_\sigma(t) = -\frac{1}{\sqrt{2\pi\sigma^3}} t \exp(-\frac{t^2}{2\sigma^2})$ as a filter to estimate the ground state energy, where σ plays a similar role to τ^{-1}. They achieved a maximal time complexity which is logarithmic in ε and a total running time $\tilde{\mathcal{O}}(\varepsilon^{-2})$. It is worth noting that for the Gaussian derivative function, when ω approaches E_j, the convolution function value will be close to zero, instead of reaching its maximum. This function is thus constrained to estimate the ground-state energy instead of transition energies. Another relevant work is by Huo and Li, which proposed the following filtering function, $f(t) = \frac{1}{\pi}\frac{\beta}{\beta^2+t^2} e^{-\frac{\beta^2+t^2}{2\tau^2}}$, a product of Lorentz and Gaussian functions [31]. However, we find that this function cannot achieve logarithmic dependence because of the sharp feature close to $t \to 0$, which results in a flat function after the Fourier transform.

Recently, there have been some related works built upon the basics of quantum mechanics that the spectral information is contained in dynamics. A representative work is the algorithmic shadow spectroscopy, which proposed to infer

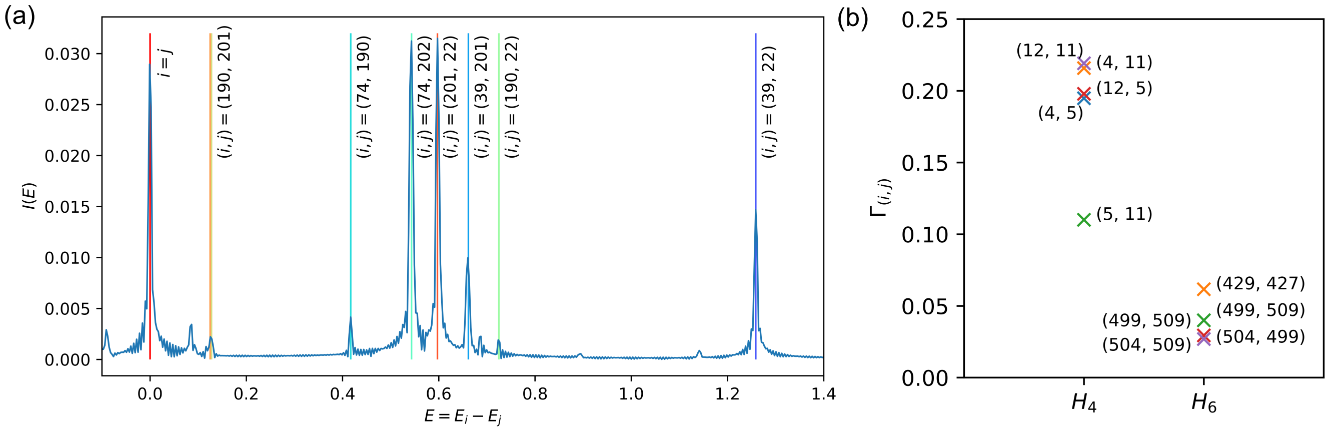

Supplementary Figure 4. (a) Transition energy spectra search of the H_4 molecule at the bond length $r = 3.0Å$. The vertical lines show ideal transition energies, which are calculated by exact diagonalisation. The observable is chosen as a particle-conserving operator $\hat{c}_5^\dagger \hat{c}_7 + \hat{c}_5^\dagger \hat{c}_7$ with fermionic (creation) annihilation operator $\hat{c}$ ($\hat{c}^\dagger$) with qubit numbering from zero. The cutoff for evaluating the integral is chosen as $T = 1$. (b) The largest coherences $\Gamma_{i,j}$ for H_4 and H_6. The transitions are labelled by a pair (i, j) aside.

the energy difference by calculating the Fourier transform of the observable expectations under time evolution [11]. These methods based on post-processing time-dependent signals inherit the problem of these classical methods, that is, generally require an evolution time proportional to the inverse of precision. By contrast, our method considers realising a spectral detector $G(\omega)$ to determine transition energies and has a theoretical guarantee for estimation accuracy. Gu *et al.* proposed an error-resilient algorithm for phase estimation without ancilla [32], similarly requiring long-time evolution. A detailed comparison between the two works could be interesting.

IV. NUMERICAL AND EXPERIMENT RESULTS

In this section, we discuss the implementation of our method in detail, and show more numerical and experimental results.

A. Transition energy estimation for molecular systems

In the main text, we showed the simulation of the energy differences for the LiH molecule, which is encoded in six qubits. The cutoff for evaluating the integral is chosen as $T = 2.5$. The observable is chosen as a particle-conserving operator $\hat{c}_0^\dagger \hat{c}_1 + \hat{c}_0 \hat{c}_1^\dagger$ with fermionic (creation) annihilation operator $\hat{c}$ ($\hat{c}^\dagger$), which considers the transitions between low-lying excited states, in order to make $\langle n|\hat{O}|n'\rangle$ non-negligible. It is anticipated that more excitations will emerge when the molecule becomes complicated. We consider H_4 and detect its excitation spectrum using an approach similar to that in the main text. Supplementary Figure 4(a) shows the excitation spectrum of H_4 at the bond length $r = 3.0Å$ encoded in eight qubits. Supplementary Figure 4(b) shows the coherence $\Gamma_{i,j}$ for H_4 and H_6.

B. Excitation spectra of lattice models

In the main text, we presented the excitation spectra of the 11-site transverse-field Ising model and the 13-site Heisenberg model. The experiment was performed on the 27-qubit IBM Kolkata quantum device. The experimental results of the real-time dynamics on the IBM device are shown in Supplementary Figure 5. We present the simulation of the energy band for the Ising and Heisenberg chains with 51 qubit in Supplementary Figure 6. We set the parameters in the same way as Figure 3 in the main text. The numerical results have a good agreement with the dispersion relations in theory.

For the Hubbard models, we find the ground state using DMRG, apply local perturbation, evolve the system under the Hamiltonian, and measure the expectation value of the number operator. The results for the bosonic chain

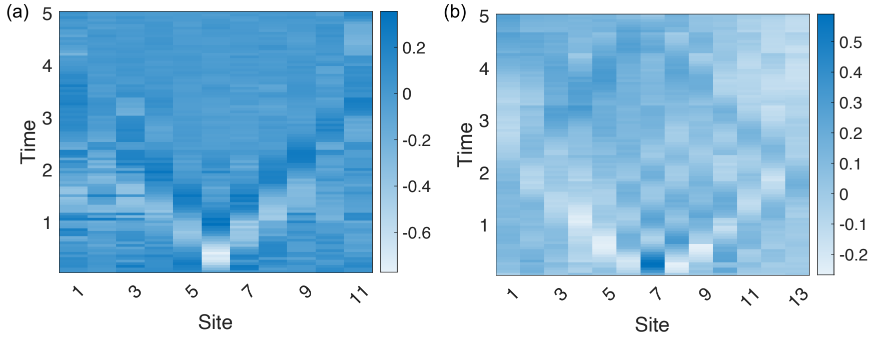

Supplementary Figure 5. (a,b) Time evolution of an 11-site transverse-field Ising model (a) and a 13-site Heisenberg mode (b) executed on the IBM Kolkata quantum device.

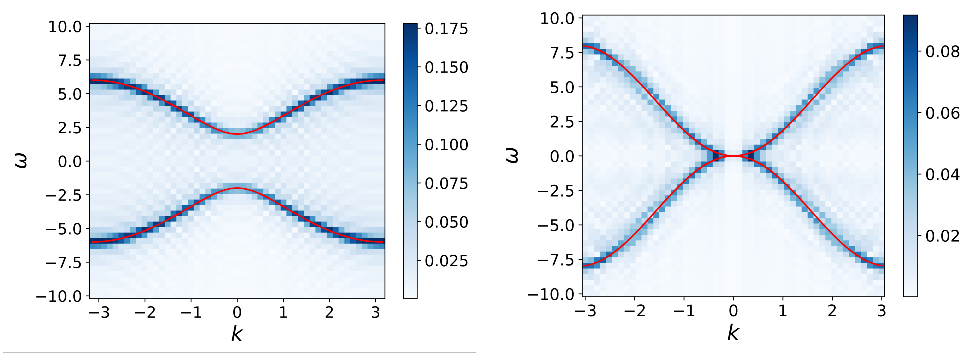

Supplementary Figure 6. Numerical simulation of the energy dispersions of lattice models. The excitation spectra of the Ising (a) and Heisenberg (b) model on a 51 site chain. The red lines represent the analytic results. The total evolution time is $T_{\text{tot}} = 10$.

with an average filling of $\bar{n} = 1.4$ and $U/J = 2$ and for the fermionic chain simulation with $h/J = 2$ are shown in Supplementary Figure 7.

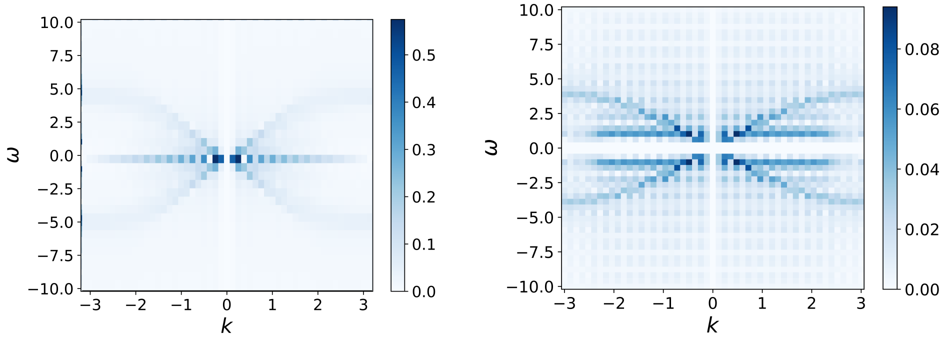

Supplementary Figure 7. Simulation of the Bose-Hubbard model for $U/J = 2$ (a), and the Fermi-Hubbard model $h/J = 2$ (b) for on a chain with $L = 49$ sites. The energy dispersion relations are shown.

Finally, we apply MPS to study the excitation spectrum of 2D lattices of dimensions $L_x \times L_y$ where $L_x = L_y = 11$. The simulation result for the lattice model with nearest-neighbour interaction is shown in Supplementary Figure 8. Even though we can obtain the spectrum of the 2D system and examine how the trend adjusts to the expected result, the resolution is limited by the significant computational complexity involved. One could improve the resolution by raising the number of sites, which, however, will significantly increase the time duration of classical simulations based on tensor networks.

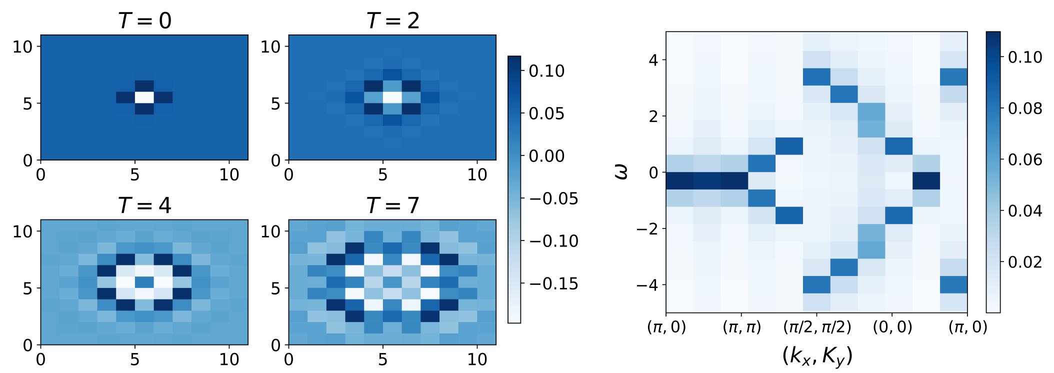

Supplementary Figure 8. Simulation of the Heisenberg model Hamiltonian applied on a square lattice with $L_x = L_y = 11$: (a) Evolution of the expectation value of the spin operator $\langle \sigma_i^y(t) \rangle$. (b) The normalized modulus of the spectral function.

[1] P. Zeng, J. Sun, X. Yuan, Universal quantum algorithmic cooling on a quantum computer. *arXiv preprint arXiv:2109.15304* (2021).
[2] E. Kökcü, H. A. Labib, J. Freericks, A. Kemper, A linear response framework for quantum simulation of bosonic and fermionic correlation functions. *Nature Communications* **15**, 3881 (2024).
[3] R. M. Nandkishore, W. Choi, Y. B. Kim, Spectroscopic fingerprints of gapped quantum spin liquids, both conventional and fractonic. *Physical Review Research* **3**, 013254 (2021).
[4] Y. Wan, N. Armitage, Resolving continua of fractional excitations by spinon echo in thz 2d coherent spectroscopy. *Physical Review Letters* **122**, 257401 (2019).
[5] G. Wang, D. Stilck-França, R. Zhang, S. Zhu, P. D. Johnson, Quantum algorithm for ground state energy estimation using circuit depth with exponentially improved dependence on precision. *arXiv preprint arXiv:2209.06811* (2022).
[6] J. Sun, X. Yuan, T. Tsunoda, V. Vedral, S. C. Benjamin, S. Endo, Mitigating realistic noise in practical noisy intermediate-scale quantum devices. *Physical Review Applied* **15**, 034026 (2021).
[7] A. A. Houck, H. E. Türeci, J. Koch, On-chip quantum simulation with superconducting circuits. *Nature Physics* **8**, 292 (2012).
[8] I. M. Georgescu, S. Ashhab, F. Nori, Quantum simulation. *Reviews of Modern Physics* **86**, 153 (2014).
[9] S. Endo, S. C. Benjamin, Y. Li, Practical quantum error mitigation for near-future applications. *Physical Review X* **8**, 031027 (2018).
[10] K. Temme, S. Bravyi, J. M. Gambetta, Error mitigation for short-depth quantum circuits. *Physical review letters* **119**, 180509 (2017).
[11] H. H. S. Chan, R. Meister, M. L. Goh, B. Koczor, Algorithmic shadow spectroscopy. *arXiv preprint arXiv:2212.11036* (2022).
[12] S. W. Lovesey, *Theory of neutron scattering from condensed matter* (1984).
[13] A. T. Boothroyd, *Principles of Neutron Scattering from Condensed Matter* (Oxford University Press, 2020).
[14] C.-K. Lee, C.-Y. Hsieh, S. Zhang, L. Shi, Simulation of condensed-phase spectroscopy with near-term digital quantum computers. *Journal of Chemical Theory and Computation* **17**, 7178–7186 (2021).
[15] C.-K. Lee, J. W. Zhong Lau, L. Shi, L. C. Kwek, Simulating energy transfer in molecular systems with digital quantum computers. *Journal of Chemical Theory and Computation* **18**, 1347–1358 (2022).
[16] Z. Jiang, K. J. Sung, K. Kechedzhi, V. N. Smelyanskiy, S. Boixo, Quantum algorithms to simulate many-body physics of correlated fermions. *Physical Review Applied* **9**, 044036 (2018).
[17] P. Jurcevic, P. Hauke, C. Maier, C. Hempel, B. Lanyon, R. Blatt, C. F. Roos, Spectroscopy of interacting quasiparticles in trapped ions. *Physical Review Letters* **115**, 100501 (2015).
[18] B. Yoshimura, W. Campbell, J. Freericks, Diabatic-ramping spectroscopy of many-body excited states. *Physical Review A* **90**, 062334 (2014).
[19] C. Senko, J. Smith, P. Richerme, A. Lee, W. Campbell, C. Monroe, Coherent imaging spectroscopy of a quantum many-body spin system. *Science* **345**, 430–433 (2014).
[20] L. Villa, J. Despres, L. Sanchez-Palencia, Unraveling the excitation spectrum of many-body systems from quantum quenches. *Physical Review A* **100**, 063632 (2019).
[21] L. Villa, J. Despres, S. Thomson, L. Sanchez-Palencia, Local quench spectroscopy of many-body quantum systems. *Physical Review A* **102**, 033337 (2020).
[22] J. Tangpanitanon, A. Megrant, Z. Chen, B. Chiaro, A. Dunsworth, *et al.*, Spectroscopic signatures of localization with interacting photons in superconducting qubits. *Science* **358**, 1175–1179 (2017).
[23] L. Villa, S. Thomson, L. Sanchez-Palencia, Finding the phase diagram of strongly correlated disordered bosons using quantum quenches. *Physical Review A* **104**, 023323 (2021).
[24] G. H. Low, I. L. Chuang, Hamiltonian simulation by uniform spectral amplification. *arXiv preprint arXiv:1707.05391* (2017).
[25] J. M. Martyn, Z. M. Rossi, A. K. Tan, I. L. Chuang, Grand unification of quantum algorithms. *PRX Quantum* **2**, 040203 (2021).
[26] A. Gilyén, Y. Su, G. H. Low, N. Wiebe, *Proceedings of the 51st Annual ACM SIGACT Symposium on Theory of Computing* (2019), pp. 193–204.
[27] H. Wang, S. Ashhab, F. Nori, Quantum algorithm for obtaining the energy spectrum of a physical system. *Physical Review A* **85**, 062304 (2012).
[28] I. Zintchenko, N. Wiebe, Randomized gap and amplitude estimation. *Physical Review A* **93**, 062306 (2016).
[29] H. Wang, Quantum algorithm for obtaining the eigenstates of a physical system. *Physical Review A* **93**, 052334 (2016).
[30] A. M. Childs, D. Maslov, Y. Nam, N. J. Ross, Y. Su, Toward the first quantum simulation with quantum speedup. *Proceedings of the National Academy of Sciences* **115**, 9456–9461 (2018).
[31] M. Huo, Y. Li, Error-resilient monte carlo quantum simulation of imaginary time. *Quantum* **7**, 916 (2023).
[32] Y. Gu, Y. Ma, N. Forcellini, D. E. Liu, Noise-resilient phase estimation with randomized compiling. *Physical Review Letters* **130**, 250601 (2023).
